# Association Between Mothers’ Life Satisfaction, Happiness and Child Discipline Practices: Evidence From a Nationally Representative Survey

**DOI:** 10.1177/08862605251353469

**Published:** 2025-07-23

**Authors:** M Mofizul Islam

**Affiliations:** 1 La Trobe University, Bundoora, Melbourne, Australia

**Keywords:** life satisfaction, happiness, disciplinary measure, children, multiple indicator cluster survey, Bangladesh

## Abstract

Mothers’ happiness, life satisfaction, and their perception of effective child-rearing can influence the disciplinary methods they employ with their children. This study examines the association between mothers’ life satisfaction, happiness, the belief in physical punishments and the disciplinary measures their children receive. Nationally representative data from the Multiple Indicator Cluster Survey Bangladesh 2019 was analyzed. The study factors were subjective happiness and life satisfaction and the belief that physical punishments are needed for the proper upbringing of their children. The outcome variables were four domains of disciplinary measures—non-violent, psychological/verbal aggression, moderate physical, and severe physical punishments—that the children experienced in the month prior to the survey. We conducted multivariable logistic and ordinal regressions. The mean happiness score for mothers was 2.99 (range: 0–4) and the life satisfaction score was 5.58 (range: 0–10). Almost 35% of mothers believed that physical punishments were necessary to raise and educate a child properly. Almost 90% of children experienced at least one form of psychological/verbal disciplinary measure. Nearly 60% experienced one or more forms of moderate physical punishment, while 30% experienced at least one form of severe physical punishment. There were significant linear negative associations between the study factors and their children receiving at least one disciplinary measure from each of the four domains. The higher the happiness or life satisfaction, the smaller the odds for children experiencing disciplinary measures from all three violent domains. Happiness and life satisfaction are determined by various personal and structural factors. Improving these factors in Bangladesh, where average national happiness is relatively low, will take time. However, prohibiting violent punishments in all settings and raising awareness of the negative impact of violent disciplinary measures on child development are achievable targets in the short term.

## Introduction

The happiness and life satisfaction of parents may substantially affect the way they rear their children. Happiness and/or life satisfaction are subjective well-being and refer to an individual’s self-evaluation of how satisfied or happy they are with their lives (Huebner, 1994). This subjective wellbeing is likely to be based on a range of factors, including health, family life, employment, social activities, recreation, consumption, ownership of properties, self, and spiritual life, marriage, the standard of living, friendship, sex life, leisure, material well-being, productivity, intimacy, safety, community and emotional well-being, money, social relationships, education, and housing (Capone et al., 2021; Ibem & Amole, 2013; Owusu Ansah et al., 2022). Individuals with lower life satisfaction are at higher risk for various psychological (Yang et al., 2023) and social problems (Kim et al., 2020). In contrast, individuals satisfied with life are more effective problem solvers and are more likely to display virtues such as forgiveness and generosity (Park, 2004).

Happiness and/or life satisfaction are associated with parenting behaviors, especially the disciplinary measures that parents exercise. This study examines the association between mothers’ life satisfaction, happiness and their belief about the necessity of physical punishments for the proper upbringing of their children and the disciplinary measures their children receive. Research indicates that when parents are satisfied or happy with their lives, they tend to engage in more positive parenting behaviors (Augustijn, 2022). Lower life satisfaction or happiness is likely to translate into lower emotional well-being and/or frustration, which may influence violent disciplinary practices such as spanking (Regalado et al., 2004). Violent disciplinary measures are linked to a range of negative outcomes for children, including physical and mental ill-health, impaired cognitive and socio-emotional development, poor educational outcomes, increased aggression and perpetration of violence (World Health Organization, 2021).

From a psychological point of view, the relationship could be explained in several ways. First, parents with low life satisfaction or happiness are more likely than their counterparts to be depressed. Although some individuals with low levels of depression may still report low life satisfaction, at the population level, multiple studies suggest that people with low life satisfaction or happiness are significantly more likely to be depressed (Koivumaa-Honkanen et al., 2004; Li et al., 2022; Seo et al., 2018). Having a relatively high degree of satisfaction and/or happiness is preventive for depressive symptoms or depression. Studies suggest that the more depressed people are, the lower their subjective well-being (Freire & Ferreira, 2020; Li et al., 2022; Ventura-Leon et al., 2022; Yildirim & Balahmar, 2020) and depressed parents are more likely than others to practice harsh disciplinary measures on their children (Kohl et al., 2011; McLearn et al., 2006). Also, low levels of life satisfaction or happiness arecharacteristically accompanied by cognitive distortions or misattributions that have been associated with deleterious parenting strategies such as harsh physical disicipline and hostility (Haskett et al., 2003; Leung & Slep, 2006; Lorber & O’Leary, 2005).

A second possible mechanism is the attachment between children and their caregivers (Ainsworth & Bowlby, 1991). The attachment theory suggests that more satisfied mothers are more sensitive and responsive to their children. Satisfied or happy mothers are likelier than unsatisfied or unhappy mothers to form secure attachments with their children (Nikolaou, 2012), which subsequently serve as a protective factor against harsh disciplinary measures (Jin et al., 2025). For instance, in their meta-analysis, Fearon et al. (2010) found that attachment insecurity was significantly linked with externalizing behaviors of children.

Finally, there is a strong positive association between mothers who practice disciplinary measures on their children and those who believe that physical punishments are needed for proper upbringing (Islam, 2024a), because belief about outcomes may justify their actions (Haney et al., 2002; Scott & Lyman, 1968). These justifications may be normative and include a trust that disciplinary measures promote obedience, teach respect, or prevent future misbehavior (Haixin et al., 2024). Beliefs about outcomes justify actions based on projected results. Therefore, extending the earlier relationships further, it can be hypothesized that mothers who have a relatively low level of life satisfaction or happiness are likely not only to use disciplinary measures but also to believe that physical punishments are needed for proper upbringing of their children.

The above-mentioned associations suggest a likely link between parents’ life satisfaction/happiness and their disciplinary practices with their children. Parents with relatively high life satisfaction/happiness are less likely to report using violent disciplinary practices in the upbringing of their children. However, to our knowledge, no study thus far has examined this relationship. Therefore, this study examines the association between mothers’ subjective measures of life satisfaction, happiness, the belief that physical punishments are needed for the proper upbringing of their children and the disciplinary measures their children receive.

## Methods

### Data

This study analyzed data from the Multiple Indicator Cluster Survey (MICS) 2019 survey in Bangladesh. MICS is an international household survey program developed by UNICEF (UNICEF, 2021). The Bangladesh Bureau of Statistics conducted the survey among women aged 15 to 49 years. MICS recruits nationally representative samples from urban and rural areas within each administrative district identified as a stratum. The sample of households was selected in two stages. In the first stage, a specified number of census enumeration areas were selected systematically with a probability proportional to the size of each stratum. Then, following a household listing within the designated enumeration zones, a random sample of 20 households was taken from each primary sampling unit in the second stage.

In the Bangladesh MICS 2019, mothers or caretakers of children under 5 years of age and of one randomly selected child aged 5 to 17 years were asked a series of questions on the methods adults in the household used to discipline the child during the past month. Complete observations on disciplinary measures were available for 49,085 children and included in the analysis of this study. The response rate in the survey was 99%. The details of the survey are available elsewhere (Bangladesh Bureau of Statistics & UNICEF-Bangladesh, 2019).

### Happiness and Life Satisfaction Variables

A natural way to measure people’s well-being is to ask them how satisfied they are with their lives. Their response to this question, for instance, on a scale of 0 to 10 allows people to evaluate their own happiness without making any assumptions about what causes it. Therefore, life satisfaction serves as a standard measure of well-being (Helliwell et al., 2023).

The independent variables (or the study factors) of this study are subjective (a) happiness, (b) overall life satisfaction, and (c) belief that physical punishments are needed to properly upbring children. The survey asked the following two questions to measure these three constructs:

“I would like to ask you some simple questions on happiness and satisfaction.

(1) First, taking all things together, would you say you are very happy, somewhat happy, neither happy nor unhappy, somewhat unhappy or very unhappy?
(2) Now, look at this ladder with steps numbered from 0 at the bottom to 10 at the top. Suppose we say that the top of the ladder represents the best possible life for you and the bottom of the ladder represents the worst possible life for you. On which step of the ladder do you feel you stand at this time?

We coded the happiness measure using a scale of 0 to 4 where 0 refers to *very unhappy* and 4 refers to *very happy*. For the life satisfaction measure, the respondents were shown a picture of a ladder with steps with discrete values between 0 and 10 and asked to identify the ladder step that represents the satisfaction level with their lives. This question is a modified version of Cantril’s ladder of life satisfaction scale (Levin & Currie, 2014). Briefly, life satisfaction is the degree to which an individual judges the overall quality of his or her life as a whole favorably (Veenhoven, 2012).

Participants’ beliefs that physical punishments are needed to properly upbring children was measured with the question “Do you believe that in order to bring up, raise, or educate a child properly, the child needs to be physically punished?” The question had three answer options: Yes, No, and Don’t know/No opinion.

### Outcome Variable

To assess the disciplinary practices children experience in the households, the MICS included a standard set of questions adapted from the Parent-Child Conflict Tactics Scale (Straus, 2017). The disciplinary questions covered physical punishment, psychological hostility, and non-violent means of discipline. Eleven disciplinary measures were listed in the questionnaire, along with questions about whether moms, caretakers, or household members had used them in the month before the study. Three of these questions dealt with non-violent methods, while the other eight addressed harsh disciplinary measures. Removing privileges, explaining why a behavior is inappropriate, and providing the youngster with an alternative activity were examples of non-violent methods. Of the eight violent disciplinary practices, the following two: (a) shouting, yelling and screaming at the child and (b) calling the child stupid, lazy or some other names are categorized as psychological or verbal aggression. The other six violent disciplinary methods are physical punishments: (a) shaking; (b) spanking; (c) hitting or slapping the child on the bottom with a bare hand; (d) hitting or slapping the child on the hand, arm, or leg; (e) hitting or slapping the child on the face, head, or ears; and (f) beating the child as hard as possible. The first four of these six harsh punishment methods can be classified as moderate, and the final two as severe. Mothers were asked whether they or any other adults in their households had used these disciplinary measures with the children in the month prior to the survey.

We used the disciplinary practices mentioned above to create three sets of indexes. The first set is binary (yes or no), which was developed based on participants’ responses regarding whether the children received at least one of the disciplinary measures from each of the four disciplinary domains. For example, a child was coded as 1 (*yes*) if they received at least one of the three non-violent disciplinary measures and 0 (*no*) if they received none of the non-violent disciplinary measures. Using this method, we developed four binary outcome variables, one for each of the four types of discipline: non-violent, psychological/verbal, moderate physical, and severe physical.

The development of the second set of outcome variables involved the utilization of three violent domains: psychosocial/verbal, moderate physical, and severe physical. In this approach, a domain was coded 1 if a child received at least one of the disciplinary measures from that domain. For example, the total domain values were 2 for a child if they received no punishment from the mild physical domain but at least one from each of the psychosocial/verbal and severe domains. Thus, the domain index ranged from 0 (i.e., received none of the psychosocial/verbal, moderate physical, or severe physical disciplinary practices) to 3 (received at least one disciplinary measure from each of the three violent disciplinary domains). We name it the summative index of violent domains.

We also developed four summative indexes using four forms of disciplinary measures: non-violent, psychological/verbal, moderate physical and severe physical. By coding 0 (for *No*) and 1 (for *Yes*) for the disciplinary measures practiced among the children, the scores for the non-violent summative index ranged from 0 to 3 (three questions). This means that if a child received none of the three disciplinary measures, the non-violent summative index for them is zero. Similarly, the psychological/verbal index ranged from 0 to 2 (two questions) and the moderate physical index ranged from 0 to 4 (four questions) and the severe physical index ranged from 0 to 2 (two questions). We name it the summative index of four types of disciplinary measures.

The mothers/caretakers were also asked if they believe that in order to bring up, raise, or educate a child properly, the child needs to be physically punished. This question had three answer options: yes, no and don’t know/no opinion. Only 0.33% of participants answered with “don’t know/no opinion.” Thus, we disregarded the “don’t know/no opinion” answer option and used the “yes” and “no” as a binary outcome.

### Analysis

We excluded children less than one year old. Descriptive analyses were performed to estimate the prevalence of the four disciplinary approaches (non-violent, psychological/verbal, moderate physical and severe physical) by a range of characteristics, including children’s age, sex, functionality, ethnicity, and residence. We conducted the following six sets of multivariable regression to examine the associations:

(1) logistic regression between life satisfaction and four disciplinary measures in terms of whether children experienced any of the non-violent, psychological/verbal, moderate physical, and severe physical punishments.
(2) logistic regression between happiness and four disciplinary measures in terms of whether children experienced any of the non-violent, psychological/verbal, moderate physical, and severe physical punishments.
(3) ordinal logistic regression between life satisfaction and the summative index developed using the three violent domains, *namely* psychological/verbal, moderate physical, and severe physical.
(4) ordinal logistic regression between happiness and the summative index developed using the three violent domains, *namely* psychological/verbal, moderate physical, and severe physical.
(5) logistic regression between life satisfaction and mothers/caretakers’ belief whether physical punishment is needed to bring up, raise, or educate a child properly.
(6) logistic regression between happiness and mothers/caretakers’ belief whether physical punishment is needed to bring up, raise, or educate a child properly.

To check the sensitivity of the association, we conducted two sets of multivariable ordinal regressions:

(1) between life satisfaction measure (as a continuous variable) and summative indexes of four forms of disciplinary measures.
(2) between happiness measure (as a continuous variable) and summative indexes of four forms of disciplinary measures.

The survey weight was applied to all analyses. For the pictorial presentation, we estimated the gradients of associations between the study factors on the outcome variables by using the post-estimation command of STATA software “margins” after running the regressions. This command computes the gradient in terms of the probability that children would be punished across the range of values of happiness and life satisfaction measures.

### Covariates

The regression models were adjusted for a range of potential confounders that were available in the dataset, including children’s sex, age, functional difficulty, ethnicity (Bengali vs. others), residence (urban and rural), mother/caretaker’s age, number of siblings, mother’s educational status, wealth quintiles of their households and administrative division. The wealth quintiles are a composite indicator of wealth. The MICS developed this variable by employing principal components analysis of information on the ownership of consumer goods, dwelling characteristics, water and sanitation, and other characteristics that are related to the household’s wealth (Bangladesh Bureau of Statistics & UNICEF-Bangladesh, 2019).

Bangladesh MICS 2019 included child functioning modules intended to provide an estimate of the number/proportion of children with functional difficulties as reported by their mothers or primary caregivers. Child functioning for children aged 5 to 17 covered questions about their ability about seeing, hearing, walking, self-care, communication, learning, remembering, concentrating, accepting change, controlling behavior, making friends, anxiety, and depression. A summary score was developed by adding the responses that produced values from 0 to 13, where 0 indicates *none* and 13 indicates the *highest* functional difficulties.

## Results

The mean age of mothers was 34.50 years (*SD*: ±6.77), and their average happiness score was 2.99 (range: 0–4; *SD*: ±0.79) and life satisfaction score was 5.58 (range: 0–10; *SD*: ±2.14; Results not shown in Table). A little more than a third (34.72%) of mothers believed that physical punishments were necessary to bring up, raise, or educate a child properly and the corresponding percentage was higher among mothers of male children (36.04%) than female children (33.34%; Table 1).

**Table 1.** Demographic Characteristics of Children and Their Mothers.

Variables	%	Mean happiness	Mean life satisfaction	Perceive the necessity of physical punishment (%)
Child’s sex				
Male	51.44	2.98	5.57	36.04
Female	48.56	2.98	5.59	33.34
Child’s age				
1–5	7.92	3.06	5.67	35.37
6–11	4.61	3.01	5.58	35.96
12–17	45.98	2.94	5.56	32.55
Child’s functional difficulty				
0	86.77	2.99	5.61	33.06
1	6.82	2.99	5.67	47.10
2	4.42	2.90	5.12	49.76
3–13	1.98	2.69	4.92	33.83
Ethnicity				
Bengali	98.89	2.99	5.58	34.72
Other	1.11	2.70	5.17	36.66
Residence				
Urban	21.44	3.05	5.93	29.63
Rural	78.56	2.97	5.48	36.15
Received at least one form of				
non-violent disciplinary measure	83.68	2.99	5.55	36.06
psychological or verbal aggression	86.73	2.99	5.55	37.59
moderate physical punishment	59.36	2.97	5.52	43.08
severe physical punishment	29.52	2.92	5.47	47.69
Mother’s age				
15–24	6.05	3.08	5.60	37.84
25–34	45.61	3.03	5.64	36.89
35–44	38.94	2.94	5.54	33.56
45+	9.40	2.88	5.45	30.83
Mother’s education level				
Pre-primary or none	25.57	2.68	4.79	35.57
Primary	27.79	2.89	5.23	37.51
Secondary	38.23	3.13	5.96	35.00
Secondary+	8.41	3.33	6.74	22.70
Household’s wealth quintiles				
Poorest	20.49	2.69	4.65	37.88
Second	21.27	2.86	5.03	38.48
Middle	19.92	3.02	5.62	36.27
Fourth	19.10	3.10	6.01	33.75
Richest	19.21	3.26	6.61	26.58
Administrative divisions				
Barisal	5.90	3.01	5.45	36.76
Chattogram	18.98	3.04	5.75	39.85
Dhaka	24.40	2.99	5.71	36.97
Khulna	11.60	2.99	6.11	36.76
Mymensingh	7.30	2.93	5.36	34.80
Rajshahi	13.33	3.05	5.33	30.66
Rangpur	11.78	2.87	5.24	37.41
Sylhet	6.71	2.91	5.08	10.41

One in four (25.57%) women had up to pre-primary level education, 27.79% had completed the primary level and 38.23% had completed education up to the secondary level (Table 1). Only 8.41% of the mothers had done higher secondary or further education. On average, their households had 2.62 children (*SD*: ±1.16).

A little more than half (51.44%) of the children were male, and 48.56% were female (Table 1). The mean age of the children was 10.95 years (*SD*: ±3.73). According to the wealth index quintile, 20.49% of the children belonged to the poorest households, 21.27% to the poorer (second quintile) households; 19.92% to the middle class (middle quintile), 19.10% to the upper-middle class and 19.21% to the households of rich quintile. Most children (78.56%) were living in rural areas and 21.44% were living in urban areas during the survey period.

Most (83.68%) mothers reported that either they or another adult in their households used at least one non-violent form of disciplinary measure upon their children during the month preceding the survey. The corresponding prevalence of any of the psychological/verbal aggressions was 86.73%, moderate physical punishments were 59.36% and severe physical punishments was 29.52%. Almost a fourth (24.30%) of mothers used at least one form of disciplinary measure from all four domains (i.e., non-violent, psychological/verbal, moderate physical, and severe punishment).

Table 2 presents the outputs of multivariable logistic regressions between life satisfaction and four types of disciplinary measures. There were significant negative associations between parental life satisfaction and four disciplinary measures that children received during the month prior to the survey. Female children were less likely to experience disciplinary measures compared to their male counterparts. However, this association was insignificant for the psychological disciplinary measure. The higher the age of the children, the less likely they were to receive any of the four disciplinary measures. Children with functional difficulties were significantly less likely to receive non-violent disciplinary measures (a*OR*: 0.94; 95% CI [0.90, 0.98]) and more likely to receive severe disciplinary measures (a*OR*: 1.15; [1.11, 1.19]). Children of other ethnic backgrounds were more likely than those of Bengali to report more non-violent, less psychological, and moderate physical disciplinary measures. Compared to children living in urban areas, those who were living in rural areas were less likely to experience psychological, moderate physical, or severe disciplinary measures. Mothers or caretakers of relatively old age were less likely than their young counterparts to report that their children experienced any of the four types of disciplinary measures. The higher the number of siblings, the more likely the children were to experience disciplinary measures, and this observation is true for all four types of disciplines. Mothers’ education appears to have a gradient relationship with four types of disciplinary measures. The higher the mothers’ educational status, the more likely they were to report their children receiving non-violent and less likely to receive psychological, moderate physical, and severe disciplinary measures.

**Table 2.** Adjusted Odds Ratios for Associations Between Life Satisfaction and Four Forms Of Disciplinary Measures That the Children Experienced in the Month Prior to the Survey.

Variables	Received at least one form of			
	Non-violent discipline, a*OR* [95% CI]	Psychological or verbal aggression, a*OR* [95% CI]	Moderate physical punishment, a*OR* [95% CI]	Severe physical punishment, a*OR* [95% CI]
Life satisfaction	0.92 [0.90, 0.93]	0.94 [0.92, 0.96]	0.98 [0.97, 0.99]	0.98 [0.97, 0.99]
Child’s sex				
Male	1	1	1	1
Female	0.91 [0.84, 0.98]	0.95 [0.88, 1.03]	0.74 [0.70, 0.79]	0.78 [0.73, 0.83]
Child’s age^§^	0.97 [0.96, 0.99]	0.91 [0.89, 0.92]	0.80 [0.79, 0.81]	0.88 [0.87, 0.89]
Child’s functional difficulty	0.94 [0.90, 0.98]	0.99 [0.94, 1.05]	1.00 [0.96, 1.03]	1.15 [1.11, 1.19]
Ethnicity				
Bengali	1	1	1	1
Other	1.67 [1.23, 2.28]	0.51 [0.39, 0.67]	0.49 [0.39, 0.63]	1.02 [0.79, 1.31]
Residence				
Urban	1	1	1	1
Rural	0.92 [0.83, 1.03]	0.87 [0.77, 0.98]	0.80 [0.73, 0.87]	0.81 [0.75, 0.88]
Mother/caretaker’s age^§^	0.99 [0.98, 0.99]	0.97 [0.96, 0.98]	0.96 [0.96, 0.97]	0.97 [0.96, 0.97]
Number of children	1.05 [1.01, 1.09]	1.12 [1.07, 1.18]	1.12 [1.08, 1.15]	1.11 [1.08, 1.15]
Mother’s educational status				
No formal education	1	1	1	1
Up to primary	1.15 [1.04, 1.28]	1.09 [0.97, 1.23]	0.97 [0.89, 1.06]	0.83 [0.75, 0.91]
Up to secondary	1.29 [1.15, 1.44]	1.05 [0.93, 1.19]	0.90 [0.82, 0.99]	0.76 [0.69, 0.84]
Higher secondary or further	1.32 [1.11, 1.57]	0.77 [0.65, 0.92]	0.70 [0.61, 0.80]	0.61 [0.53, 0.71]
Household’s wealth index				
Poor	1	1	1	1
Lower middle	1.14 [1.02, 1.26]	1.05 [0.93, 1.19]	0.96 [0.88, 1.05]	0.98 [0.90, 1.07]
Middle	1.21 [1.08, 1.35]	1.09 [0.96, 1.24]	0.97 [0.88, 1.07]	0.97 [0.88, 1.06)
Higher middle	1.21 [1.07, 1.37]	0.99 [0.86, 1.15]	0.83 [0.75, 0.92]	0.92 [0.83, 1.02]
Rich	1.48 [1.27, 1.72]	0.96 [0.81, 1.13]	0.66 [0.58, 0.74]	0.79 [0.69, 0.89]
Administrative division				
Barisal	1	1	1	1
Chattogram	0.9 [0.78, 1.05]	2.33 [1.99, 2.73]	1.72 [1.52, 1.94]	1.39 [1.22, 1.59]
Dhaka	1.03 [0.89, 1.20]	2.27 [1.95, 2.65]	1.82 [1.62, 2.06]	2.69 [2.37, 3.05]
Khulna	1.73 [1.47, 2.03]	2.71 [2.31, 3.19]	1.66 [1.48, 1.88]	1.94 [1.71, 2.21]
Mymenshing	0.65 [0.55, 0.77]	1.75 [1.45, 2.11]	1.29 [1.11, 1.49]	2.08 [1.78, 2.42]
Rajshahi	0.60 [0.52, 0.69]	1.94 [1.66, 2.28]	0.97 [0.86, 1.09]	1.33 [1.16, 1.53]
Rangpur	0.77 [0.67, 0.90]	2.66 [2.25, 3.15]	1.54 [1.36, 1.75]	1.48 [1.30, 1.70]
Sylhet	0.47 [0.40, 0.56]	1.66 [1.37, 2.02]	1.46 [1.26, 1.70]	1.15 [0.98, 1.36]

§ as a continuous variable.

**Figure 1.**
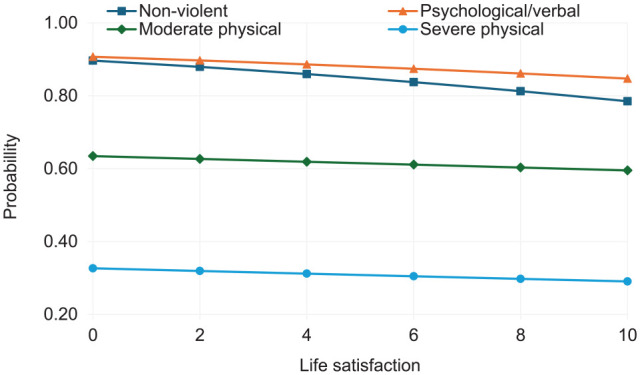
Margin plots presenting the probabilities for children experiencing four forms of disciplinary measures across the levels of life satisfaction of their mothers.

Figure 1 presents the margin plots developed after running the multivariable logistic regressions between life satisfaction and four types of disciplinary measures. There are almost linear gradient relationships between the life satisfaction and probabilities of children experiencing psychological/verbal, moderate physical and severe physical punishments. However, the margins plot for non-violent disciplinary measures is slightly curvy.

Table 3 presents the outputs of multivariable logistic regressions between happiness (as a continuous measure) and four types of disciplinary measures. The findings were largely consistent with those observed for the relationship between life satisfaction and the four disciplinary measures. Margin plots indicate a linear relationship between these two study factors (i.e., life satisfaction and happiness) and the severe form of disciplinary measures.

**Table 3.** Adjusted Odds Ratios for Associations Between Happiness and Four Forms of Disciplinary Measures That the Children Experienced in the Month Prior to the Survey.

Variables	Received at least one form of			
	Non-violent discipline, a*OR* [95% CI]	Psychological or verbal aggression, a*OR* [95% CI]	Moderate physical punishment, a*OR* [95% CI]	Severe physical punishment, a*OR* [95% CI]
Happiness	0.85 [0.81, 0.90]	0.88 [0.83, 0.93]	0.89 [0.86, 0.93]	0.86 [0.82, 0.89]
Child’s sex				
Male	1	1	1	1
Female	0.91 [0.84, 0.97]	0.95 [0.87, 1.03]	0.74 [0.70, 0.78]	0.78 [0.73, 0.82]
Child’s age	0.97 [0.96, 0.99]	0.91 [0.89, 0.92]	0.80 [0.79, 0.81]	0.88 [0.87, 0.89]
Child’s functional difficulty	0.94 [0.90, 0.98]	0.99 [0.94, 1.05]	0.99 [0.96, 1.03]	1.15 [1.11, 1.19]
Ethnicity				
Bengali	1	1	1	1
Other	1.63 [1.20, 2.21]	0.50 [0.38, 0.65]	0.49 [0.38, 0.62]	1.01 [0.78, 1.30]
Residence				
Urban	1	1	1	1
Rural	0.92 [0.82, 1.02]	0.87 [0.77, 0.98]	0.81 [0.74, 0.88]	0.82 [0.76, 0.89]
Mother/caretaker’s age	0.99 [0.98, 0.99]	0.97 [0.96, 0.98]	0.96 [0.96, 0.97]	0.96 [0.96, 0.97]
Number of children	1.06 [1.01, 1.10]	1.13 [1.08, 1.18]	1.12 [1.08, 1.16]	1.11 [1.08, 1.15]
Mother’s educational status				
No formal education	1	1	1	1
Up to primary	1.16 [1.04, 1.29]	1.09 [0.97, 1.24]	0.98 [0.90, 1.07]	0.84 [0.77, 0.93]
Up to secondary	1.28 [1.14, 1.43]	1.05 [0.92, 1.19]	0.92 [0.84, 1.01]	0.79 [0.72, 0.87]
Higher secondary or further	1.29 [1.09, 1.53]	0.77 [0.64, 0.92]	0.72 [0.63, 0.82]	0.65 [0.56, 0.75]
Household’s wealth index				
Poor	1	1	1	1
Lower middle	1.13 [1.02, 1.26]	1.06 [0.94, 1.19]	0.98 [0.89, 1.07]	1.00 [0.92, 1.09]
Middle	1.17 [1.05, 1.31]	1.08 [0.95, 1.23]	0.99 [0.90, 1.09]	1.00 [0.91, 1.10]
Higher middle	1.14 [1.01, 1.29]	0.97 [0.84, 1.12]	0.85 [0.77, 0.94]	0.95 [0.86, 1.06]
Rich	1.37 [1.17, 1.59]	0.93 [0.79, 1.09]	0.67 [0.60, 0.76]	0.82 [0.73, 0.93]
Administrative division				
Barisal	1	1	1	1
Chattogram	0.89 [0.77, 1.04]	2.31 [1.98, 2.70]	1.70 [1.50, 1.91]	1.37 [1.21, 1.57]
Dhaka	1.04 [0.89, 1.21]	2.27 [1.94, 2.65]	1.80 [1.60, 2.03]	2.64 [2.33, 3.00]
Khulna	1.65 [1.41, 1.94]	2.62 [2.23, 3.09]	1.63 [1.44, 1.84]	1.90 [1.67, 2.16]
Mymensingh	0.65 [0.55, 0.77]	1.75 [1.45, 2.11]	1.29 [1.11, 1.49]	2.07 [1.77, 2.42]
Rajshahi	0.62 [0.53, 0.72]	1.98 [1.69, 2.32]	0.97 [0.86, 1.10]	1.34 [1.17, 1.53]
Rangpur	0.77 [0.67, 0.90]	2.66 [2.25, 3.14]	1.53 [1.35, 1.73]	1.46 [1.27, 1.67]
Sylhet	0.49 [0.41, 0.58]	1.69 [1.39, 2.06]	1.46 [1.25, 1.69]	1.14 [0.97, 1.34]

Table 4 presents the results of multivariable ordinal logistic regressions where the study factor was life satisfaction or happiness and the outcome variable was the summative index developed using the three violent domains. The second column of the table shows the model outputs with life satisfaction as the study factor, while the third column presents the outputs with happiness as the study factor. For a one-unit increase in life satisfaction, the odds for children experiencing disciplinary measures from all three violent domains versus the combined none to two violent domains were 2% lower, when all other variables in the model are held constant. Likewise, the odds for children experiencing disciplinary measures from all three violent or at least two of the violent domains versus the combined none to only one domain were 2% lower, when all other variables in the model are held constant. In the model with happiness as the study factor, the corresponding odds are 11% lower (a*OR*: 0.89; 95% CI [0.86, 0.91]). The results for the covariates such as children’s sex, age and functionality were mostly similar to those found in the ordinary logistic regressions mentioned above.

**Table 4.** Adjusted Odds Ratios for the Associations Between Life Satisfaction/Happiness and the Summative Index Developed Using the Three Violent Domains.

Variables	Summative index developed using the three violent domains, a*OR* [95% CI]	
Life satisfaction	0.98 [0.97, 0.99]	—
Happiness	—	0.89 [0.86, 0.91]
Child’s sex		
Male	1	1
Female	0.81 [0.77, 0.84]	0.80 [0.77, 0.84]
Child’s age	0.87 [0.86, 0.88]	0.87 [0.86, 0.88]
Child’s functional difficulty	1.10 [1.06, 1.13]	1.09 [1.06, 1.13]
Ethnicity		
Bengali	1	1
Other	0.77 [0.64, 0.94]	0.77 [0.64, 0.93]
Residence		
Urban	1	1
Rural	0.84 [0.79, 0.89]	0.84 [0.79, 0.89]
Mother/caretaker’s age	0.97 [0.97, 0.97]	0.97 [0.97, 0.97]
Number of children	1.10 [1.08, 1.13]	1.10 [1.08, 1.13]
Mother’s educational status		
No formal education	1	1
Up to primary	0.91 [0.85, 0.97]	0.93 [0.87, 0.99]
Up to secondary	0.86 [0.80, 0.92]	0.88 [0.82, 0.94]
Higher secondary or further	0.70 [0.64, 0.78]	0.73 [0.66, 0.80]
Household’s wealth index		
Poor	1	1
Lower middle	0.99 [0.93, 1.05]	1.00 [0.94, 1.07]
Middle	0.98 [0.92, 1.05]	1.00 [0.94, 1.07]
Higher middle	0.91 [0.85, 0.98]	0.93 [0.86, 1.00]
Rich	0.79 [0.72, 0.85]	0.81 [0.74, 0.88]
Administrative division		
Barisal	1	1
Chattogram	1.51 [1.38, 1.65]	1.49 [1.37, 1.63]
Dhaka	2.12 [1.94, 2.32]	2.09 [1.91, 2.28]
Khulna	1.77 [1.62, 1.93]	1.73 [1.58, 1.89]
Mymensingh	1.62 [1.44, 1.81]	1.61 [1.43, 1.81]
Rajshahi	1.21 [1.10, 1.32]	1.21 [1.11, 1.33]
Rangpur	1.55 [1.41, 1.69]	1.53 [1.39, 1.67]
Sylhet	1.27 [1.15, 1.42]	1.27 [1.14, 1.41]

Table 5 presents the output of logistic regressions between life satisfaction or happiness (as a study factor) and the perceived necessity of physical punishment to properly bring up, raise or educate children. Overall, the results are similar to those in the other regression models presented earlier. For each unit increase in life satisfaction, the odds for women to perceive that physical punishment is needed to properly bring up, raise or educate children reduced by 2% (a*OR*: 0.98; 95% CI [0.96, 0.99]. The corresponding reduction in odds per unit increase in happiness measure was 9% (a*OR*: 0.91; [0.87, 0.95]). The odds ratios for the covariates, such as children’s sex and age, were similar to those found in other regression models presented above.

**Table 5.** Adjusted Odds Ratios for the Associations Between Life Satisfaction/Happiness and the Perception of Mothers That Physical Punishment is Necessary to Properly Raise a Child.

Variables	Physical punishment is needed to properly bring up, raise or educate a child, a*OR* [95% CI]	
Life satisfaction	0.98 [0.96, 0.99]	—
Happiness	—	0.91 [0.87, 0.95]
Child’s sex		
Male	1	1
Female	0.89 [0.83, 0.95]	0.89 [0.83, 0.95]
Child’s age	0.98 [0.97, 0.99]	0.99 [0.97, 0.99]
Child’s functional difficulty	1.07 [1.03, 1.12]	1.07 [1.03, 1.11]
Ethnicity		
Bengali	1	1
Other	0.90 [0.66, 1.23]	0.89 [0.65, 1.22]
Residence		
Urban	1	1
Rural	1.16 [1.06, 1.29]	1.17 [1.06, 1.29]
Mother/caretaker’s age	0.98 [0.97, 0.99]	0.98 [0.97, 0.99]
Number of children	1.08 [1.04, 1.12]	1.08 [1.04, 1.12]
Mother’s educational status		
No formal education	1	1
Up to primary	1.02 [0.92, 1.12]	1.02 [0.93, 1.13]
Up to secondary	0.92 [0.83, 1.02]	0.93 [0.84, 1.03]
Higher secondary or further	0.62 [0.53, 0.74]	0.63 [0.54, 0.75]
Household’s wealth index		
Poor	1	1
Lower middle	1.00 [0.90, 1.11]	1.01 [0.91, 1.11]
Middle	0.94 [0.85, 1.05]	0.95 [0.85, 1.06]
Higher middle	0.89 [0.79, 0.99]	0.90 [0.80, 1.01]
Rich	0.70 [0.61, 0.81]	0.71 [0.62, 0.82]
Administrative division		
Barisal	1	1
Chattogram	1.22 [1.06, 1.40]	1.21 [1.05, 1.39]
Dhaka	1.20 [1.04, 1.37]	1.18 [1.03, 1.36]
Khulna	1.06 [0.92, 1.21]	1.04 [0.90, 1.19]
Mymenshing	0.93 [0.78, 1.10]	0.92 [0.79, 1.10]
Rajshahi	0.78 [0.68, 0.90]	0.79 [0.68, 0.90]
Rangpur	1.05 [0.92, 1.21]	1.04 [0.91, 1.20]
Sylhet	0.21 [0.16, 0.26]	0.21 [0.16, 0.26]

The results of the sensitivity analysis are presented in Table 6. Overall, the odds ratios both for life satisfaction and happiness measures are consistent with those found in other regression models presented above. The reductions in odds of summative indexes for all four types of disciplinary measures are higher for each unit increase in happiness than the increase in life satisfaction measure. This means that the higher the life satisfaction or happiness, the less likely that the children in those households experience disciplinary measures.

**Table 6.** Results of Sensitivity Analysis Presenting Adjusted Odds Ratios for Children Experiencing Four Forms of Disciplinary Measures.

Variables	Summative index for four forms of disciplinary measures			
	Non-violent, a*OR* [95%CI]	Psychological or verbal aggression, a*OR* [95% CI]	Moderate physical a*OR* [95% CI]	Severe physical a*OR* [95% CI]
Life satisfaction	1.00 [0.99, 1.01]	0.98 [0.97, 0.99]	0.98 [0.97, 0.99]	0.98 [0.97, 0.99]
Happiness	0.95 [0.92, 0.98]	0.90 [0.87, 0.94]	0.91 [0.88, 0.93]	0.87 [0.84, 0.91]

## Discussion

The results of this study suggest that mothers who report higher levels of life satisfaction or happiness are less likely to resort to disciplinary measures with their children. The impact of happiness on disciplinary measures appears to be stronger than that of life satisfaction. Additionally, the study suggests that mothers with higher levels of life satisfaction or happiness are less inclined to believe that physical punishment is necessary for proper child-rearing. These three relationships show a consistent linear pattern. Overall, the findings underscore the importance of parental wellbeing in reducing the harmful disciplinary practices children experience. These findings are of significant public health importance, as violent disciplinary methods can be detrimental to children’s healthy development (Islam, 2024b), and a considerable number of mothers report lower levels of happiness or life satisfaction, with a substantial proportion believing in the necessity of physical discipline for raising children.

The adjusted odds ratios for the happiness measure were much smaller than those for life satisfaction. This is likely to be because the happiness measure has only five categories (*very unhappy*, *somewhat unhappy*, *neither happy nor unhappy*, *somewhat happy*, *very happy*). After coding these five categories, happiness as a continuous variable had a value range of 0 to 4. In comparison, life satisfaction has a range of 0 to 10. Consequently, for the life satisfaction measure, the change in odds was computed for a relatively smaller unit compared to the happiness measure. As a result, the denominator is bigger for per larger unit of happiness than it was for smaller unit of happiness.

In addition to the theoretical explanations presented in the introduction, there may be other factors to consider. For instance, disruptive or inappropriate behaviors of children can cause relatively low parental life satisfaction or happiness (Nelson et al., 2014). Also, at a population level, parents of children who display disruptive or inappropriate behaviors are more likely than others to practice harsh disciplinary practices when all other factors remain constant (Woodward & Fergusso, 2002). Further studies are recommended to investigate these relationships more thoroughly.

In Bangladeshi culture, there is a strong tradition of using disciplinary punishments in child-rearing. As a result, Bangladesh has a greater rate of violent punishment than most of its neighboring countries. For example, 86% of children aged 1 to 14 reported experiencing psychological aggression in the context of discipline from a mother (Bangladesh Bureau of Statistics & UNICEF-Bangladesh, 2019). This is in contrast to roughly 77% in Nepal, 73% in Pakistan, and 61% in Afghanistan (Fry et al., 2020). Although parents or other adults of the family find justifications for violent disciplinary practices and often implicate them in child education, in many cases, violent punishment is an outlet to vent their frustrations rather than an attempt to educate children (Leung & Slep, 2006). The gradient effect of associations we found in this study further corroborates that part of the disciplinary punishments that the children experience is attributed to mothers’ happiness or life satisfaction, rather than children’s misdeeds that may warrant a disciplinary measure as per the social custom.

Several covariates were found to be significantly associated with the outcome variables. Of them, positive associations for a child’s functionality and the number of children in the household are likely to be related to parenting stress. Additional parenting tasks related to managing children with disabilities, behavior management difficulties, and parenting without a benchmark because typical developmental milestones may not be applicable standards for children with disabilities, etc., in turn, might increase parents’ stress and likelihood of responding harshly to the child. Similarly, the relatively more time and efforts required to rear multiple children is also stressful. For instance, Anthony et al. (2005) found that parents who reported greater parenting stress tended to acknowledge using more harsh discipline, such as corporal punishment.

We observed a clear correlation between the educational attainment of mothers and the disciplinary actions taken toward their children. Additionally, mothers with higher education were less inclined to believe that physical punishment is necessary for proper child-rearing. It is likely that these mothers act according to their belief (Haney et al., 2002; Scott & Lyman, 1968). In particular, the inverse and gradient relationship between the study factors and the outcomes emphasizes the significance of girls’ education. Bangladesh has made significant strides in its efforts to provide basic education during the last three decades, particularly among girls. Nevertheless, there is still much work to be done to address the high dropout rate of girls from secondary education. Despite initiatives like the Female Secondary Stipend and Assistance, which have notably enhanced girls’ retention at the secondary level, the dropout rate remains as high as 40% (Bangladesh Bureau of Educational Information and Statistics [BANBEIS], 2022).

The findings of this study have some policy implications. According to the World Happiness Report 2023, based on a 3-year-average (2020–2022), Bangladesh ranks 118th among 137 UN member states for which data were available on happiness assessment. Clearly, as per this population-level measurement, Bangladesh is sitting at the lower end of the ranking. However, data are not available separately for men and women. Since physical and mental health, income and employment, social support and personal freedom are some of the key factors of happiness or life satisfaction, women are likely to have lower happiness or life satisfaction than their male counterparts. Even if a fraction of violent disciplinary measures children experience is attributed to mothers’ happiness/life satisfaction, at a population level the magnitude of harm for children is substantial. As per the existing law in Bangladesh, all forms of corporal punishment in educational institutions are prohibited but not in the home environment (End Violence Against Children & End Corporal Punishment, 2022; Ministry of Education, 2011). The government should immediately prohibit violent punishments in all settings. Undoubtedly, improving parental life satisfaction, happiness and changing the existing perception about the necessity of physical punishment for the children are difficult to achieve. However, at least raising awareness of the adverse effects of violent disciplinary measures on child development and the importance of positive parenting are achievable tasks. Concerned ministries should actively promote this awareness-building task. Evidence from some other countries suggests that violent disciplinary practices are likely to substantially decrease when relevant laws and public awareness-related education are consistently implemented (Bussmann et al., 2011).

### Limitations

The study has several limitations. First, it is a cross-sectional study, which means that it captures a snapshot of data at a specific point in time, and thus the associations are correlational only. Second, the study asked mothers whether they or other adults in their households had used disciplinary measures. As a result, the relationships observed in this study are associations between the mothers’ subjective measures of life satisfaction or happiness and the types of disciplinary measures children in those households experienced. This means we cannot definitively conclude that mothers with higher life satisfaction or happiness are less likely than their counterparts to use violent disciplinary measures with their children. However, the consistency of findings about mothers’ perception of the necessity of physical punishment in properly raising children suggests that we could have drawn this conclusion if we had specific data about the disciplinary measures they themselves used. Third, some of these children may have received disciplinary punishments because they display violent behavior. A significant negative association in those contexts may mean that mothers of those children have relatively low life satisfaction or happiness, when other factors are held constant. This means that the association could be bidirectional, as we stated in a previous paragraph. Longitudinal data are needed to understand this relationship. Also, data only allow us to count the types of disciplinary measures used during the month preceding the survey. The mothers might not clearly remember all the disciplinary measures they or the other adults of the households exercised. However, the effect of such a bias is likely to be random. Additionally, data were collected in 2019, so the findings may not reflect the current situation. The impact of COVID-19 and the subsequent cost-of-living crisis could have influenced overall life satisfaction and happiness. Increasing awareness of adverse health outcomes of violent disciplinary measures may have led to a decrease in their prevalence over the years. These changes suggest that the observed associations might differ if a more recent dataset were available. At last, but not least, the study may have encountered social desirability bias because the respondents may not have reported all disciplinary measures, particularly violent practices towards their children.

## Conclusion

Mothers’ perceived happiness and life satisfaction are significantly associated with their children experiencing disciplinary measures. A similar association was found between their happiness/life satisfaction and the perception that physical punishment is needed to properly bring up, raise or educate children. These negative associations are linear for the three violent disciplinary measures, namely psychological/verbal, moderate physical and severe physical punishments. Consistently, the higher the happiness or life satisfaction, the smaller the odds for children experiencing disciplinary measures from all three violent domains. Happiness and life satisfaction are well-being issues determined by various personal and structural factors. Improving these factors in a country like Bangladesh, where average national happiness is relatively low, will take time. However, taking steps to prohibit violent punishments in all settings and promoting awareness of the negative impact of violent disciplinary measures on child development and the importance of positive parenting areachievable in the short run.
